# The impact of complications following open colectomy on hospital finances: a retrospective cohort study

**DOI:** 10.1186/2047-0525-3-1

**Published:** 2014-03-07

**Authors:** David N Flynn, Rebecca M Speck, Najjia N Mahmoud, Guy David, Lee A Fleisher

**Affiliations:** 1 Department of Anesthesiology and Critical Care, Perelman School of Medicine, University of Pennsylvania, 3400 Spruce Street, Philadelphia, PA 19104, USA; 2 Center for Pharmacoepidemiology Research and Training, Center for Clinical Epidemiology and Biostatistics, Perelman School of Medicine, University of Pennsylvania, 423 Guardian Drive, Philadelphia, PA 19104, USA; 3 Department of Surgery, Hospital of the University of Pennsylvania, 3400 Spruce Street, Philadelphia, PA 19104, USA; 4 Department of Health Care Management, The Wharton School, University of Pennsylvania, Colonial Penn Center, 3641 Locust Walk, Philadelphia, PA 19104, USA; 5 Senior Fellow, Leonard Davis Institute of Health Economics, University of Pennsylvania, Colonial Penn Center, 3641 Locust Walk, Philadelphia, PA 19104, USA

**Keywords:** Cost, Complications, Colectomy

## Abstract

**Background:**

When hospitals suffer financial losses when postoperative complications occur, they may have a direct financial incentive to initiate quality improvement programs. The purpose of this research was to determine the relationship between complications following open colectomy and hospital finances.

**Methods:**

After obtaining Institutional Review Board approval, we conducted a retrospective chart review of 276 open colectomies performed at the Hospital of the University of Pennsylvania. The medical records were manually reviewed for complications that occurred within 30 days after surgery. Financial information, including total, fixed and variable costs, was obtained from the hospital’s cost accounting database. Reimbursement assuming payment by Medicare was calculated. Differences in costs, reimbursements and total margins were analyzed.

**Results:**

Of 276 patient records reviewed, 61 (22%) of the patients experienced postoperative complications. When complications occurred, mean total costs increased from $23,101 to $48,180, fixed costs increased from $14,516 to $30,339 and variable costs increased from $8,535 to $17,848 (*P* < 0.001 for each comparison); the mean reimbursement increased from $23,231 to $35,651 (*P* < 0.001); and the total margin decreased from $131 to - $12,528 (*P* < 0.001). Complications were associated with a more than twofold increase in length of stay in the hospital. Multiple regression modeling indicated similar increases in each of the financial variables and length of stay as a result of postoperative complications. The impact of these complications on each outcome measure was similar in effect for patients in the matched subset of 100 patients.

**Conclusion:**

Our results demonstrate a financial incentive for hospitals to investigate quality improvement measures to prevent postoperative complications and avoid the associated financial losses.

## Background

Value is the new focus of the American health-care system [1], with payers, providers and policy makers searching for ways to improve outcomes and contain costs [2]. Complications following surgery are value-destroying events because they contribute to poor outcomes while increasing the costs borne by hospitals and health-care payers [3]. As a result, initiatives designed to prevent surgical complications, such as the National Surgical Quality Improvement Program (NSQIP), have gained increasing importance at both the individual hospital and nationwide levels [4].

The impact of improved health-care quality on hospital finances is of great importance to hospital financial managers and administrators. Launching an initiative to reduce surgical complications requires allocation of scarce hospital resources, potentially at the expense of alternative quality improvement programs [5,6]. Quantifying the impact of postoperative complications on hospital resources is therefore a useful exercise that can help financial managers optimally allocate these resources to benefit both patients and hospitals.

Colectomy has become a focus of quality improvement initiatives because of the large number of these operations performed each year and the high incidence of associated complications. The procedure, performed on nearly 275,000 patients annually [7], has been shown in several studies to have 30-day complication rates exceeding 25% [8-10]. A 2008 study of 36 general surgical procedures involving more than 129,000 patients found that colectomy, though it represented less than 10% of all cases, was responsible for 24.3% of all adverse events and the greatest share of morbidity, mortality and increased length of stay (LOS) of the procedures studied [9]. Furthermore, a majority of adverse events involving colon resection are considered preventable [11], suggesting that quality improvement applied to colon surgery could be a particularly fruitful undertaking.

Although numerous studies have demonstrated that surgical complications significantly increase hospital costs, data regarding the overall financial impact of postoperative complications on hospitals are scarce. Although some of these additional costs may be offset by higher reimbursement from payers [3,12], little is known about the sources of increased hospital costs or the extent to which payers compensate hospitals for increased expenses for postoperative complications.

We hypothesized that surgical complications would be financially detrimental to hospitals, resulting in increased costs and decreased profit margins. In this study, we examined the increment in hospital costs and reimbursements associated with complications following open colectomies at a large academic medical center. We modeled reimbursement under the simplifying assumption that all patients were insured by Medicare. Using cost and reimbursement data, we calculated total margins (that is, profitability) and compared the results between patients who experienced complications and patients who did not. Finally, we discussed the implications of our results on the financial case for complication prevention from the perspective of hospital financial managers and administrators.

## Methods

### Study design and setting

We conducted a retrospective review of all medical records of patients who underwent open partial colectomy with anastomosis during a 4-year period (2007 through 2010) at the Hospital of the University of Pennsylvania, a large, urban academic medical center. This study was conducted after we received approval from the Institutional Review Board of the University of Pennsylvania. Approval included a waiver of documentation of informed consent, given the retrospective, minimal risk nature of the research. The study was conducted and reported in accordance with the Strengthening the Reporting of Observational Studies in Epidemiology statement for observational studies [13].

### Data sources

Patient data and dates of admissions were obtained from the hospital’s administrative database. Complications were identified by manual review of the hospital’s electronic medical records. Financial data were obtained from the hospital’s internal cost accounting database (Horizon Performance Manager Cost Accounting System; McKesson, San Francisco, CA, USA). The accounting system assigns a unit cost to each charge item or service provided (excluding physicians’ professional fees) using either the relative value unit (RVU) or ratio of cost to charge (RCC) cost accounting methods. The RCC method is used to determine the costs of medications and supplies, and the RVU method is used to assign costs to particular services, from both service centers (for example, nursing or radiology) and support centers (for example, information technology or housekeeping) [7]. The costs for each item or service provided during an encounter are then mapped to charge categories (fixed costs, variable labor costs and variable supply costs), which are then added to determine the total cost of resources consumed during each encounter.

### Participants

Study patients were located by searching the hospital’s administrative database for Current Procedural Terminology (CPT) code 41440 (open partial colectomy with anastomosis). Study exclusion criteria included (1) age younger than 18 years, (2) concomitant surgery involving an organ outside the lower gastrointestinal (GI) tract, (3) failure to perform surgery within 48 hours after hospital admission and/or (4) incomplete medical or financial records. Demographic data collected for each patient included age, gender, American Society of Anesthesiologists (ASA) physical status score and body mass index (BMI).

### Complications

Each patient’s electronic medical record was manually reviewed for major complications occurring within 30 days after surgery. Complications abstracted included deep wound surgical site infection (SSI), organ/space infection SSI, sepsis, *Clostridium difficile* colitis, pneumonia, myocardial infarction, new-onset arrhythmia, cardiac arrest, pulmonary embolism, acute renal failure and/or acute kidney injury, cerebrovascular accident, hemorrhage requiring transfusion of more than 4 U of packed red blood cells (RBCs), bowel obstruction, reoperation, death and other complications. *Other complications* included any condition not present upon admission that could potentially be harmful to the patient’s health that was documented in the medical record by an attending physician. We used standard American College of Surgeons NSQIP definitions when available. *C. difficile* colitis and bowel obstruction were recorded on the basis of the attending physician’s diagnosis.

### Financial outcomes and lengths of stay

Clinical data were merged with financial data to determine the costs associated with each patient’s care. Costs examined included total costs, fixed costs and variable costs. For patients who were readmitted to the hospital within 30 days after the initial surgery, the cost of each inpatient encounter was added to determine the total cost for all hospital admissions. All costs were adjusted to 2010 US dollars (USD) using the hospital inpatient services subcategory of the Consumer Price Index (CPI) obtained from the US Bureau of Labor Statistics [12].

The patients in this study were insured by various payers, including Medicare, Medicaid and commercial insurers, so, in addition to obtaining the actual payments, we calculated expected reimbursements under the simplifying assumption that all study patients were insured by Medicare only. This step was included to eliminate the impact of payer mix on financial outcomes, which may vary substantially between hospitals and have a significant impact on reimbursement and overall profitability. We calculated the expected Medicare reimbursement for each patient using the Medicare Severity Diagnosis-Related Group (MS-DRG) payment rate at our hospital during each year of services. (A brief overview of the Medicare system is provided in the Appendix). A unique MS-DRG was assigned for each admission using an automated MS-DRG grouper based on International Classification of Disease, Ninth Revision (ICD-9) discharge and procedure codes (3M Co, St Paul, MN, USA). Payment rates for 2007 were not available, so the corresponding 2008 rates were used for patients treated during 2007 and 2008. Payments were adjusted to 2010 USD using the general CPI. LOS was also calculated for each patient. For patients who were readmitted within 30 days after surgery, LOS from the index visit and subsequent readmissions occurring within the study period were added to determine the total LOS associated with the procedure.

### Statistical methods

Bivariate comparisons of continuous data were made using Student’s *t*-test. The impact of complications on financial variables was analyzed using multiple linear regression analysis. Additional explanatory variables used in the regression analysis included age, age-squared (to allow for nonlinear effects of age on the rate of complications), gender, ASA classification and BMI.

To control for potential confounding effects resulting from underlying differences between groups, we used the Coarsened Exact Matching (CEM) algorithm [14] to match patients who experienced complications on a one-to-one basis to patients without complications. Patients were matched for age, gender, ASA classification and BMI. CEM is relatively a new nonparametric matching method developed to address limitations of more widely used methods such as propensity score matching. CEM has been described in detail elsewhere, so we will discuss it here only briefly. When performing CEM, the original values of the covariates are first coarsened, or divided into two or more user-defined ranges or categories. The CEM algorithm is then used to create a set of strata, each of which contains variables that share identical coarsened values for every covariate. Strata that contain patients in both the treatment and control groups are retained, and patients from the treatment group and control group paired within each stratum. After matching has been performed, the coarsened values are replaced by their original values, allowing subsequent analysis of the uncoarsened values of each covariate to proceed.

We used CEM because it offers several advantages over existing methods, such as Propensity Score Matching (PSM). First, CEM is a member of the Monotonic Imbalance Bounding (MIB) class of matching methods, which generalizes the Equal Percent Bias Reducing (EPBR) class (comprising PSM and many other popular matching methods, such as Mahalanobis matching, Genetic Matching and others), eliminating key assumptions required to generate unbiased estimates of treatment effects. Second, with CEM, the process used to create matches is reversed. With CEM (and MIB methods in general), the user chooses the maximum level of imbalance *ex ante* (determined by the level of coarsening), with the number of matches produced as a result. With EPBR methods, the number of matched observations is chosen *ex ante*, with an unknown amount of imbalance produced as a result. Thus, with CEM, the need for repeated postmatch balance-checking, model modification and rematching required by other methods is eliminated, although the level of coarsening may need to be altered to produce a satisfactory sample size. Third, with CEM, the imbalance on one covariate can be changed in isolation without affecting the imbalance on any other covariates. This contrasts with EPBR methods, in which altering the imbalance on one covariate changes the imbalance on all other covariates, sometimes in unpredictable ways. Fourth, CEM has been shown through simulation on multiple data sets to be superior to alternative matching methods by producing matches with reduced imbalance, bias, variance, model dependence, estimation error and mean square error [15,16].

All statistical analyses were conducted using Stata version 11.0 software (StataCorp, Austin, TX, USA). For all calculations, statistical significance was defined by *P*-values less than 0.05.

## Results

A total of 380 patients were identified, 276 of whom met our inclusion criteria. Reasons for exclusion included concomitant surgery outside the lower GI tract (47 patients), surgery more than 48 hours after admission (48 patients) and incomplete medical or financial records (3 patients). An additional six patients met two exclusion criteria because they had concomitant surgery outside the GI tract more than 48 hours after admission.

Among the 276 included patients, 61 patients (22% of the total) experienced one or more major complications (Table 1). The most common complication was organ/space SSI (5.1% of patients), followed by new-onset arrhythmia (4.7%), reoperation (4.7%) and bowel obstruction (4.0%). All other complications were relatively uncommon, occurring in fewer than 3.0% of patients. Only one patient death occurred, which followed the only recorded cardiac arrest (believed to be precipitated by a massive pulmonary embolism). Postoperative complications were often related. For example, 9 of the 13 reoperations were performed as a result of either deep SSI or organ and/or surgical space infections.

**Table 1:** **Number and incidence of complications**^**a**^

**Complications**	**Number of patients**	**%**
Deep SSI	5	1.8%
Organ/space SSI	14	5.1%
Sepsis	5	1.8%
*Clostridium difficile* colitis	4	1.4%
Pneumonia	8	2.9%
Bowel obstruction	11	4.0%
Acute kidney injury	6	2.2%
Cerebrovascular accident	2	0.7%
Pulmonary embolism	4	1.4%
New-onset arrhythmia	13	4.7%
Myocardial infarction	5	1.8%
Cardiac arrest	1	0.4%
Unplanned intubation	6	2.2%
Reoperation	13	4.7%
Death	1	0.4%
Other infections	7	2.5%

^a^SSI: Surgical site infection. The category “Other infections” included readmission for unexplained severe gastritis, hypotension of unknown cause prompting transfer to the surgical intensive care unit (SICU), upper-extremity ischemia presumed to be caused by vasospasm, respiratory depression from opiate overdose requiring SICU transfer, readmission for biliary colic requiring surgical intervention, readmission for chylous ascites requiring paracentesis and postdural puncture headache requiring epidural blood patch (*n* = 1 for each complication).

### Unmatched sample

There were no statistically significant differences between patient groups with respect to age, gender, BMI or ASA classification in the unmatched samples (Table 2). However, patients in the complications group tended to be older, more often male and had higher ASA scores than those in the uncomplicated group. Mean LOS was more than twice as long for patients who experienced complications (15.6 days) than for those who had no complications (7.3 days).

**Table 2:** **Patient demographic data, length of stay and financial data by complication group for matched and unmatched samples**^**a**^

	**Entire cohort (*N*** **= 276)**				**Matched sample (*n*** **= 100)**			
**Variable**	**Uncomplicated**	**Complications**	***P*-value**	**Difference**	**Uncomplicated**	**Complications**	***P*-value**	**Difference**
Patients, *n* (%)	215 (78%)	61 (22%)	–	–	50	50	–	–
Mean age, years (SD)	60.2 (14.8)	62.9 (13.3)	0.20	2.7	63.6 (12.9)	63.7 (12.3)	0.97	0.1
Females, *n* (%)	113 (53%)	27 (44%)	0.25	–	24 (48%)	24 (48%)	1.0	–
ASA class, mean (SD)	2.5 (0.5)	2.6 (0.5)	0.21	0.1	2.6 (0.50)	2.6 (0.50)	1.0	0
BMI, mean (SD)	28.4 (7.3)	28.7 (6.3)	0.74	0.3	28.1 (6.4)	28.2 (5.7)	0.93	0.1
LOS (days), mean (SD)	7.3 (2.4)	15.6 (7.8)	<0.001	8.3	7.4 (2.1)	15.9 (7.5)	<0.001	8.5
Total cost, mean (SD)	$23,101 (6,914)	$48,180 (26,596)	<0.001	$25,079	$23,381 (6,712)	$49,733 (28,279)	<0.001	$26,351
Fixed cost, mean (SD)	$14,516 (4,252)	$30,229 (16,340)	<0.001	$15,713	$14,807 (4,509)	$31,233 (17,333)	<0.001	$16,426
Variable cost, mean (SD)	$8,535 (2,904)	$17,848 (10,356)	<0.001	$9,313	$8,526 (2,656)	$18,397 (11,066)	<0.001	$9,871
Reimbursement, mean (SD)	$23,231 (6,761)	$35,651 (13,445)	<0.001	$12,420	$23,632 (7,110)	$35,739 (13,327)	<0.001	$12,108
Total margin, mean (SD)	$131 (8,309)	-$12,528 (26,113)	0.001	-$12,659	$250 (7,781)	-$13,993 (27,144)	<0.001	-$14,244

^a^ASA: American Society of Anesthesiology; BMI: Body mass index. Total margin = Reimbursement - Total cost. Financial data are US dollars.

Complications were associated with dramatic increases in total, fixed and variable costs; increased reimbursements; and decreased total margins (Table 2). The mean total cost was $23,101 for patients in the uncomplicated group compared to $48,180 for those in the complications group, a difference of $24,079. Fixed and variable costs were more than doubled when complications occurred. Mean reimbursement was $12,420 higher for patients in the complications group ($35,651) than for patients in the uncomplicated group ($23,231). Patients in the uncomplicated group generated a small but positive total margin (that is, gain) of $131 per patient, whereas patients in the complications group generated a large negative total margin (that is, loss) of $12,528 per patient.

Table 3 displays the results of the multiple linear regression analysis for each outcome variable, adjusted for the effects of age, age-squared, gender, ASA classification and BMI. The results of this analysis were similar to the unadjusted differences between the two groups with regard to the mean values (Table 2). Complications were associated with an increase in total cost of $24,889, an increase in reimbursement of $12,326 and a decrease in total margin of $12,563.

**Table 3:** **Relationships between major surgical complications and cost, reimbursement, total margin and length of stay, controlling for effects of age, gender, American Society of Anesthesiology score and body mass index**^**a**^

	**Entire patient cohort (*N*** **= 276)**						**Matched sample (*n*** **= 100)**					
**Dependent variable**	**Total cost**	**Fixed cost**	**Variable costs**	**Reimbursement**	**Total margin**	**Length of stay (days)**	**Total cost**	**Fixed cost**	**Variable costs**	**Reimbursement**	**Total margin**	**LOS (days)**
Complication	$24,889^b^	$15,610^b^	$9,228^b^	$12,326^b^	-$12,563^b^	8.29^b^	$26,269^b^	$16,368^b^	$9,846^b^	$12,138^b^	-$14,131^b^	8.456^b^
	(2,024)	(1,246)	(799)	(1,272)	(2,083)	(0.607)	(4,168)	(2,566)	(1,634)	(2,175)	(4,022)	(1.116)
Age	-$118	-$54	-$63	-$153	-$35	-0.135	$854	$572	$281	$16	-$838	0.256
	(393)	(242)	(155)	(247)	(404)	(0.118)	(1,498)	(922)	(587)	(782)	(1,445)	(401)
Age-squared	$1	$1	$1	$1	$0	0.001	-$8	-$6	-$3	$0	$8	-0.002
	(3)	(2)	(1)	(2)	(3)	(0.001)	(12)	(7)	(5)	(6)	(12)	(0.003)
Female gender	$2,047	$1,258	$796	-$578	-$2,625	0.493	$4,442	$2,698	$1,759	-$991	-$5,432	1.093
	(1,680)	(1,034)	(663)	(1,056)	(1,729)	(0.504)	(4,313)	(2,655)	(1,691)	(2,251)	(4,161)	(1.154)
ASA score	$2,598	$1,397	$1,200	$1,391	-$1,207	0.611	$2,272	$1,194	$1,074	-$159	-$2,430	0.411
	(1,758)	(1,082)	(694)	(1,104)	(1,809)	(0.528)	(4,755)	(2,927)	(1,864)	(2,482)	(4,588)	(1.272)
BMI	$53	$38	$16	-$110	-$163	0.006	-$51	-$17	-$34	-$208	-$158	-0.024
	(121)	(75)	(48)	(76)	(125)	(0.036)	(375)	(231)	(147)	(196)	(362)	(0.100)
Constant	$15,636	$9,791	$5,797	$27,531^b^	$11,895	8.011^c^	-$2,900	-$2,330	-$584	$31,214	$34,114	0.494
	(11,860)	(7,298)	(4,682)	(7,450)	(12,202)	(3.559)	(45,162)	(27,804)	(17,704)	(23,573)	(43,578)	(12,090)^c^
Observations	276	276	276	276	276	276	100	100	100	100	100	100
*R*^2^	0.3764	0.3835	0.3509	0.2733	0.1391	0.4290	0.3137	0.3197	0.2945	0.2600	0.1496	0.3928

^a^ASA: American Society of Anesthesiology; BMI: Body mass index; LOS: Length of stay. ^b^*P* < 0.001. ^c^*P* < 0.05. All values were obtained using multiple linear regression techniques as described in the Methods section. Values in parentheses are standard errors.

### Matched sample

The 50 patients in who experienced complications were matched to 50 similar patients who without complications. Suitable matches were not found for the remaining 11 patients who had complications. Differences in age, gender, BMI and ASA classification were minimal between complication groups in the matched sample (Table 2).

Complications were associated an increase in total costs of $26,351, an increase in reimbursement of $12,108 and a decrease in total margin of $14,244. Complications were associated with an increase in LOS of 8.5 days.

The results of the multivariate regression analysis (Table 3) were nearly identical to the unadjusted results. After adjustment, complications were associated with an increase in total cost of $26,269, an increase in reimbursement of $12,138 and a decrease in total margin of $14,131.

## Discussion

The purpose of this study was to evaluate the financial impact of surgical complications following open colectomy from the perspective of hospital financial managers and administrators. To eliminate the impact of payer mix on our results, we used the simplifying assumption that all patients included in the study were insured by Medicare. We found that major surgical complications were associated with higher total hospital cost and reimbursement, but decreased profitability, under Medicare payment. When complications occurred, cost increased from $23,101 to $48,180, reimbursement increased from $23,231 to $35,651 and profitability fell from a gain of $131 to a loss of $12,528.

There were no statistically significant differences in demographics or health-related attributes between study groups. This suggests that patient baseline health status cannot explain the cause or risk of complications observed. However, there were minor, non–statistically significant differences between our study groups. Patients who experienced major complications tended to be older, were assigned a higher preoperative ASA classification and were more likely to be male. Each of these factors could independently impact cost and profitability. We used a powerful matching algorithm to generate nearly perfectly matched study groups to control for these effects. Interestingly, the financial impact of major complications was even greater between the matched study groups than the original unmatched groups. This provides strong support for the assertion that the observed differences in financial outcomes were driven by the occurrence of complications rather than by variability between groups.

We used multivariate linear regression to evaluate the impact of complication status, age/age-squared, ASA classification, BMI and gender on cost, reimbursement, profitability and LOS. Only complication status had a statistically significant impact on outcomes, increasing total cost by $24,889, increasing reimbursement by $12,326 and decreasing profitability by $12,563. The results of multivariate analyses performed on the matched sample were nearly identical. This further supports the argument that complication status, rather than other factors, independently drives financial performance and strongly suggests that hospitals could benefit financially by preventing complications.

LOS has an important but underappreciated impact on hospital finances. Any event that prolongs LOS generates an opportunity cost for hospitals, especially as the health care environment is moving toward bundled payment [17,18]. Whenever a patient’s LOS is prolonged, the hospital’s available bed capacity is decreased, thus limiting the hospital’s ability to admit additional revenue-generating patients. The cost of a missed admission is equal to the expected revenue minus the expected variable cost (that is, the contribution margin). Because variable costs make up a small component of a hospital’s total costs, the contribution margin will nearly always be large and positive. We found that complications more than doubled the LOS, indicating that the hospital could treat two uncomplicated patients in the same time required to treat single patients who experienced a major complication. Thus, our data indicate that hospitals suffer financially in at least two ways when complications occur: first, through lower expected profit margins due to treating patients who experience complications, and second, via the opportunity cost generated when capacity is consumed by prolonged hospital LOS.

Medicare has recently started to deny payment for selected diagnoses when readmissions occur within 30 days of discharge, including congestive heart failure, myocardial infarction and pneumonia [19]. This policy will eventually be extended to include other conditions, including readmissions related to surgical complications. For the purposes of this study, we assumed that all readmissions were reimbursed fully by Medicare. Denial of payment for readmissions would result in losses greater than those calculated in this study, thus providing additional incentives for hospitals to prevent complications and the readmissions that frequently accompany them.

Eappen and coauthors [17] found that surgical complications were associated with increased total margin for commercially insured patients but decreased total margin for patients who were self-insured or covered by Medicare or Medicaid. Owing to the relatively small sample size in our study, we assumed that all patients were insured by Medicare, thus eliminating the impact of payer mix on our results. In contrast to the findings of Eappen *et al*., however, when we used actual payment received in the analysis, regardless of payer type, patients who experienced complications generated negative margins (losses averaging nearly $8,000/patient), whereas uncomplicated patients generated positive margins (gains of nearly $1,200/patient). This suggests that the financial incentive to prevent complications is preserved when actual payment is considered at our institution.

In this study, we did not evaluate the impact of individual types of complications on financial performance or the preventability of different complications. Previous studies have demonstrated that certain complications are far more expensive to treat than others [20,21]. Additionally, certain complications may be far more difficult to prevent than others, whereas some complications may simply be inevitable because of the underlying disease burden of surgical patients. From a strictly financial standpoint, treatment of patients with complications that are both preventable and associated with the highest cost should be prioritized.

This study has several limitations that warrant discussion. First, physicians’ professional fees and payments were not included in the analyses, so our results may understate both the increase in cost and reimbursement associated with complications. Second, because our chart review was limited to a single hospital, patients whose readmissions occurred at other hospitals were not included in our analysis. Although it may have been feasible for us to contact all study patients and inquire about readmissions elsewhere, we used retrospective data in this study. The only potentially link of patients to this study would have been direct contact for a research telephone call, which they did not consent to at the time of procedure. Third, although we modeled reimbursement by assuming all patients were insured through Medicare, our study population might not be representative of the Medicare population. The average age of patients in each complication group, for example, was slightly less than the minimum age of 65 years at which patients become eligible for Medicare benefits. The fourth limitation concerns the generalizability of this study to other settings. All of the data in this study derived from a single US institution, with its own unique cost structure and patient population. Thus, it is difficult to predict how accurately the cost increases found in this study would predict changes in cost associated with complications at other hospitals. Moreover, hospitals operating outside of the United States generate revenue based on the financing systems of their home countries, which may differ significantly from the MS-DRG rates used in this study to determine reimbursement and calculate margins. In many countries, however (for example, England, France and Germany) the methods used to derive rates (or tariffs) share many similarities to Medicare’s MS-DRGs, including adjustment for patients’ comorbidities, complications and local market forces [22]. Therefore, we can predict that both costs and payments in these settings would increase when complications occur, but additional research is required to determine the extent to which payment increases offset increased expenditures.

## Conclusions

Our results provide a strong argument for financial investment in quality improvement from the perspective of the hospital administrator. Increases in Medicare reimbursement resulting from postoperative complications are not large enough to compensate the hospital for the increase in total costs associated with complications. Prevention of surgical complications therefore can help to improve care quality while improving cost-effectiveness.

## Appendix: the US Medicare system

Medicare is a social health insurance program administered by the US Department of Health and Human Services. Eligible beneficiaries include Americans ages 65 or older as well as Americans of any age with certain chronic diseases. Medicare is a large source of payment for inpatient hospital care in the United States, comprising 27% of all acute care hospital revenue in 2012 [23].

Medicare payment is broken into different components, each of which pays for different aspects of medical care. Medicare Part A (hospital insurance) pays for inpatient hospital care, home health care, skilled nursing facilities and hospice care. Medicare Part B (supplementary medical insurance) pays for outpatient physician and nursing services, diagnostic imaging and testing, and outpatient hospital procedures. Medicare Part D (prescription drug plan) is a voluntary, premium-based program that helps to subsidize the cost of prescription drugs for Medicare beneficiaries.

Inpatient hospital care (Medicare Part A) is financed by the Medicare inpatient prospective payment system. Each hospital is paid a predetermined amount per discharge based on the beneficiary’s clinical condition and treatment strategy, which are captured through MS-DRGs. The payment rates for each MS-DRG are updated each year in an attempt to account for the average cost of resources required to treatment patients in that MS-DRG. Payments for individual hospitals are further adjusted on the basis of geographic differences with regard to labor prices, costs of providing graduate medical education and compensation of hospitals that provide care to a disproportionate share of low-income patients.

The MS-DRG system consists of 335 base DRGs, which are split into two or three MS-DRGs on the basis of the presence of one or more complications or comorbidities (CCs) or major CCs. For example, a healthy patient who undergoes a colectomy with no resulting complications may be assigned MS-DRG 331 (major small- or large-bowel procedures without CCs), whereas a patient with multiple comorbidities may be assigned MS-DRG 330 (major small- or large-bowel procedures with CCs) and a patient who experiences a serious complication may be assigned MS-DRG 329 (major small- or large-bowel procedure with major CCs). Accordingly, the hospital would receive the highest payment for MS-DRG 329, less for MS-DRG 330 and the least for MS-DRG 331.

## Abbreviations

ASA: American Society of Anesthesiologists; BMI: Body mass index; CEM: Coarsened Exact Matching; CPI: Consumer Price Index; CPT: Current Procedural Terminology; EPBR: Equal Percent Bias Reducing; ICD-9: International Classification of Diseases Ninth Revision; LOS: Length of stay; MIB: Monotonic Imbalance Bounding; MS-DRG: Medicare Severity Diagnosis-Related Group; NSQIP: National Surgical Quality Improvement Program; PSM: Propensity score matching; RBC: Red blood cell; RCC: Ratio of cost to charge; RVU: Relative value unit; SSI: Surgical site infection; USD: US dollars.

## Competing interests

The authors declare that they have no competing interests.

## Authors’ contributions

DF participated in the study design, completed data collection and drafted the manuscript. RS participated in study design and coordination and performed the statistical analysis. NM participated in the study design and helped to draft the manuscript. GD participated in the study design and performed the statistical analysis. LF conceived the study, participated in its design and coordination, and helped to draft the manuscript. All authors read and approved the final manuscript.
